# Affective Dimensions in Maternal Voice During Child Feeding in Mothers With and Without Eating Disorder History—Findings From a Machine Learning Analysis of Speech Data

**DOI:** 10.1002/erv.70038

**Published:** 2025-10-03

**Authors:** Jana Katharina Throm, Manuel Milling, Andreas Triantafyllopoulos, Alexander Kathan, Annica Franziska Dörsam, Johanna Löchner, Björn Schuller, Katrin Elisabeth Giel

**Affiliations:** ^1^ Department of Psychosomatic Medicine and Psychotherapy University Hospital Tuebingen Tuebingen Germany; ^2^ Centre of Excellence for Eating Disorders Tuebingen (KOMET) University Hospital Tuebingen Tuebingen Germany; ^3^ EIHW—Embedded Intelligence for Health Care and Wellbeing University of Augsburg Augsburg Germany; ^4^ CHI—Chair of Health Informatics Technical University of Munich MRI Munich Germany; ^5^ MCML—Munich Center for Machine Learning Munich Germany; ^6^ Department of Child and Adolescent Psychology and Psychotherapy Friedrich‐Alexander‐University Erlangen‐Nuremberg Erlangen Germany; ^7^ German Center for Mental Health (DZPG) Tübingen Germany; ^8^ MDSI—Munich Data Science Institute Technical University of Munich Munich Germany; ^9^ GLAM—Groupon Language, Audio & Music Imperial College London UK

**Keywords:** eating disorder, machine learning, mealtime, mother‐child‐communication, speech analysis

## Abstract

**Objective:**

Eating disorder (ED) history may impact mother‐child communication during mealtimes and contribute to transgenerational transmission of ED. This study employed machine learning (ML) to identify speech characteristics during mother‐child feeding interactions, aiming for investigating whether vocalised affective characteristics differ between mothers with and without ED history when feeding their child.

**Method:**

Mothers with (*n* = 17) and without ED history (*n* = 27) and their children (10 months) were filmed at home during mealtime. Various ML models were exploratively tested to assess their suitability for analysing maternal voice data. Diagnosis of an ED history was based on the structured Eating Disorder Examination Interview.

**Results:**

A ML model specialised for the prediction of emotional arousal, valence and dominance provided the most pronounced differences between the groups. These variables were consistently stronger expressed in the voices of mothers with ED history during child feeding, predominantly in the middle of the interaction.

**Conclusions:**

Voice data suggests that mothers with ED history might be emotionally stronger involved throughout child feeding. This indicates that there are differences in communication between women with and without ED history and highlights the importance of research into maternal communication in affected families. ML approaches are promising tools as they can detect more subtle nuances compared to questionnaires.

## Highlights

- Differences in affective dimensions of the voices of mothers with and without ED history were found, highlighting the need for further research to explore the role of communication in the transmission of ED.
- Mothers with ED history expressed stronger emotionality in their voices throughout child feeding, with ‘emotional hotspots’ generally occurring in the middle of the feeding interaction.
- ML approaches are promising tools as they can detect more subtle nuances compared to questionnaires.

## 1 Introduction

Eating disorders (ED) such as anorexia nervosa (AN), bulimia nervosa (BN) and binge‐eating disorder (BED) are serious and complex mental disorders which may transmit to following generations (Bould et al. 2015). Maternal ED are associated with problematic feeding behaviour, difficulties in children's cognitive development, and a higher risk of mental disorders in the offspring (Martini et al. 2020; Watson et al. 2018). Furthermore, numerous studies reported an increased risk for the development of ED in children of affected mothers (Bould et al. 2015; Lydecker and Grilo 2017; Ziobrowski et al. 2019), with both, genetic and environmental factors as potential underlying mechanisms (Martini et al. 2020).

In recent years, there has been increasing interest in the role of communication in the intergenerational transmission of disordered eating (Arroyo et al. 2017; Oliveira et al. 2019). A cross‐sectional study was conducted to investigate the relationship between current eating behaviour of women (aged 18–40 years) and early caregiver eating messages (Oliveira et al. 2019). Correlation analysis indicated a significant association between the recall of restrictive/critical caregiver eating messages and disordered eating (Oliveira et al. 2019). Furthermore, maternal commentary about weight and size was found to be a significant mechanism for the transmission of EDs in a three generation study (Arroyo et al. 2017). Parent‐child interaction was further found to be severely affected in parents with depression and a key aspect in the transition of depression for example long‐term effects of risk and resilience factors in families with children aged 0–3 in a representative sample (Dubber et al. 2015). However, there is little evidence on the impact of maternal ED history on mother‐child communication during mealtimes, therefore, a major research gap remains regarding this issue (Chapman et al. 2021; Throm, Schilling, et al. 2024).

Outside the mealtime context, one study reported that maternal AN diagnosis was associated with emotionally unmatched flat mother‐child dialogues lacking involvement and interest of mothers and children (Cimino et al. 2020). Furthermore, mothers with ED history were reported to use more verbal control in the interaction with their children during play and mealtime (Stein et al. 1994, 2001). Most of the few studies investigating verbal mother‐child mealtime communication in mothers with ED history date back to the 1990ies and report more negative expressed emotion toward their infants (Stein et al. 1994) and fewer positive comments about food and eating (Waugh and Bulik 1999) compared to controls. However, in a more recent study by our group, we found negligible interaction differences between mothers with and without during child feeding, relying on a structured coding scheme (Doersam et al. 2024).

Previous analyses of speech in persons with ED were mainly based on simple counting or rating procedures and bear the risk of subjective judgement. In contrast, using technology‐supported digital analysis approaches may help to overcome these difficulties and greatly expand the possibilities for the analysis of speech data. Automated identification of diseases using artificial intelligence on speech data is rapidly emerging in recent years (Milling, Pokorny, et al. 2022; Berardi et al. 2023). Beyond ED, the analysis of emotional states ‐ including emotional dimensions such as valence, arousal, and dominance ‐ has raised a fair amount of interest for investigating aspects of mental disorders, such as depression or autism, especially in computationally‐aided approaches (Valstar and et al. 2013; Milling, Baird, et al. 2022). By modelling data using machine learning (ML) approaches, it may be possible to detect differences in speech, that remain hidden using other analysis methods. ML has been previously used in the context of EDs, with promising outcomes regarding the ability to predict ED status and the forecast of symptom progression (Fardouly et al. 2022; Levinson et al. 2023). Therefore, applying ML approaches on videotaped mealtime situations of mother‐child dyads with and without maternal ED history may allow to draw further conclusions on the impact of communication on adverse child outcomes in families with ED history.

The main objective of this study was to investigate the possibility of distinguishing mothers with and without an ED history based on their voice recordings during a videotaped feeding situation with their child. This was achieved through exploratory testing of different ML models, which analysed the voices of mothers including the dimensions of valence, arousal and dominance.

1. We hypothesised that mothers with ED history can be distinguished from mothers without ED history through ML voice analysis during child feeding at the end of the first year postpartum.
2. Furthermore, we hypothesised that mothers with ED history would differ from mothers without ED history regarding emotional dimensions in maternal voice during child feeding at the end of the first year postpartum.

## 2 Method

### 2.1 Study Design and Participants

**FIGURE 1:**
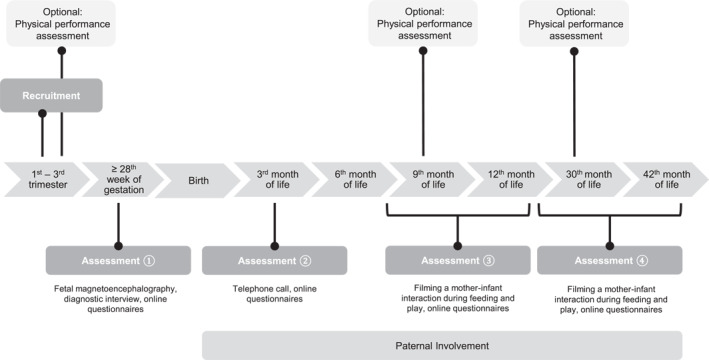
EMKIE study procedure (Doersam et al. 2024).

The data used in this study stem from the EMKIE study (Doersam et al. 2024, 2022; Dörsam et al. 2022; Throm, Dörsam, et al. 2024), a family cohort study conducted at the Department of Psychosomatic Medicine and Psychotherapy at the Medical University Hospital Tübingen. The EMKIE study assesses the influence of maternal ED on offspring and the family system from late pregnancy (≥ 28th week of pregnancy) until 42 months postpartum, distributed over four measurement points. An overview of the EMKIE study procedure is provided in Figure 1.

Women with and without ED history according to DSM‐5 criteria, including AN, BN, BED and other specified feeding or eating disorder (OSFED), and their partners were recruited in pregnancy between 2018 and 2022. ED history was evaluated with the Eating Disorder Examination‐Interview (EDE) (Hilbert and Tuschen‐Caffier 2016) administered in late pregnancy by a trained staff member, using an adapted version for the assessment of previous ED. Inclusion criteria were an age between 18 and 40 years, an inconspicuous singleton pregnancy and birth and sufficient German language skills. Women with a severe medical illness that require acute treatment and make study participation difficult or impossible were excluded. Exclusion criteria on the child's part comprised chromosomal abnormalities, congenital infections and severe perinatal complications. Recruitment was carried out using announcements in parenting magazines, e‐mails in the university and clinic mailing list, flyers at gynaecologist's offices, and contacting former ED patients treated at the University Hospital Clinic.

### 2.2 Procedures

This paper focuses on data from assessment 3 of the EMKIE study, which was conducted between eight and 12 months postpartum. A study member visited the families in their homes at the child's regular mealtime. Prior to the meal, time was scheduled to complete questionnaires. This was to allow the child and the mother to become acquainted with the situation and the presence of the study staff, thereby reducing potential behavioural biases during the filming of the meal. Following this brief period of acclimatization, the feeding interactions of the mother‐infant dyads were recorded using a smartphone mounted on a tripod positioned on the table. There were no guidelines on the type of food or how the mother should feed her child. During the mealtime, the study member left the room where possible, in order to minimise distractions and ensure that the situation was as natural as possible. The filming continued until the mother announced the end of the meal. Some families recorded a meal by themselves, as in some cases, a home visit was not possible due to corona restrictions or due to long travel distances. Furthermore, the mothers were required to complete online questionnaires regarding their eating behaviours, perceived stress, and depressive symptoms. Further details on the longitudinal results of the questionnaires can be found elsewhere (Throm, Dörsam, et al. 2024).

### 2.3 Measures

*Eating Disorder Examination‐Questionnaire.* The German Version of the Eating Disorder Examination‐Questionnaire (EDE‐Q) was used to assess severity of ED psychopathology (Hilbert, Tuschen‐Caffier, et al. 2016). The four subscales *Restraint, Eating Concern, Weight Concern and Shape Concern* with a total of 22 items ascertain the intensity of ED psychopathology of the last 28 days. Six further items record diagnostically relevant behaviour. Frequencies and intensity of ED‐specific characteristics are rated on seven‐point Likert‐scales. Higher scores represent more severe ED psychopathology. Internal consistency is high (*α* ≤ 0.97) and the questionnaire is sensitive to change (Hilbert, Tuschen‐Caffier, et al. 2016).

*Perceived Stress Scale*. A German Version (Klein et al. 2016) of the Perceived Stress Scale (PSS10) (Cohen et al. 1983) was used to measure participants' perceived stress. The 10‐item self‐report scale indicates the level of subjective stress of the last month, measured on 5‐point Likert‐scales ranging from 0 (never) to 4 (very often). The German Version demonstrated good internal consistency (*α* = 0.84) and construct validity (Klein et al. 2016).

*Patient Health Questionnaire‐9.* The depression module of the Patient Health Questionnaire (PHQ‐D) was applied to screen for depressive symptomatology (Löwe and et al. 2001). Each of the nine items is scored on a four‐point Likert‐scale. The score varies from 0 to 27, with a score lower than 5 indicating the absence of a depressive disorder (Löwe and et al. 2001). The PHQ‐D has been reported to have excellent criterion validity and good internal consistency for depressive disorders (*α* = 0.88) (Gräfe et al. 2004).

At study inclusion, the women completed a self‐report questionnaire to assess maternal sociodemographic data. Further information regarding pregnancy were extracted from the medical record of prenatal and natal care.

### 2.4 Ethics

This study was approved by the ethics committee of the medical faculty of the Eberhard‐Karls‐University and the University Hospital Tübingen (219/2018BO1; 859/2021BO2). All study participants analysed in this study provided written informed consent.

### 2.5 Statistical Analysis

IMB SPSS Statistics for Windows, Version 28.0 was used for statistical analysis. Fisher's exact test was used to assess intergroup differences in nominal data. Metric data was checked for normal distribution and intergroup differences calculated with Student's *t*‐test and Mann‐Whitney‐*U*‐test, depending on the result. Level of significance was defined as *p* < 0.05. Analysis of variance (ANOVA) was used to assess group differences with regard to the emotional dimensions.

### 2.6 Video Data Analysis With Machine Learning Approaches

The main goal of our ML analyses was the attempt to train a classifier that can distinguish between individuals with and without ED, solely based on their voice recordings. Therefore, we first resampled the raw audio signal before applying SpeechBrain (Ravanelli et al. 2021) in order to detect speech and remove non‐speech segments like silence or ambient noise from the audio. This allowed us to automatically segment the data into *utterances*—segments of continuous speech without (long) pauses. These utterances formed our units of analyses, that is, the ‘instances’ over which the following feature extraction steps and ML algorithms were performed. Overall, we used two different procedures for extracting features over each utterance:

- *Rolling‐window features*, where the features were extracted over overlapping windows. This means that for an input utterance of duration X and a window ‘hop’ of Y, voice parameters were extracted X/Y times. This process is further outlined in (Miranda and et al. 2022).
- *Utterance‐level features*, where the features were extracted over the entire utterances. This means that for an input utterance of duration X, voice parameters were extracted once for the entire duration X of the audio. This is a standard process that has been used in multiple previous works for speech analysis.

For (a), we extracted the extended Geneva Minimalistic Acoustic Parameter Set (eGeMAPS), a standardized expert‐selected set of 88 acoustic high level descriptors (Eyben et al. 2015) and the Mel frequency cepstral coefficients (MFCCs), a representation of the short‐term energy spectrum on the human‐hearing‐inspired Mel‐scale (Davis and Mermelstein 1980). eGeMAPS, in particular, has proven to be very effective on a variety of voice‐based tasks (Triantafyllopoulos et al. 2023), such as depression (Milling, Pokorny, et al. 2022; Gerczuk et al. 2023), PTSD (Kathan et al. 2023), or stress (Baird et al. 2021).

For (b), we extracted eGeMAPS as well as learnt representations from large, pretrained deep neural networks. Specifically, we utilised models derived from the state‐of‐the‐art wav2vec2.0 architecture (Baevski and et al. 2020), namely w2v2‐large, a model trained on a large English dataset, and w2v2‐emo (Wagner et al. 2023), a variant of w2v2‐large which was further fine‐tuned to predict arousal, valence, and dominance on a large, speech emotion recognition dataset in a normalised range from 0 to one in each emotional dimension. For the last model in particular, we experimented both with its intermediate representations (w2v2‐emo‐emb) and its output predictions (w2v2‐emo‐avd), the latter having the added benefit of being *interpretable* as proxies for emotional arousal, valence, and dominance.

Finally, for the ML steps of the training, we applied random forests (RFs) and support vector machines (SVMs) to find decision boundaries between the two classes. The individuals of the study were separated into 5 subject‐independent folds, which were balanced with respect to eating disorder condition and control group. In other words, all data of each of the 44 subjects was assigned to one of the five folds, leading roughly nine subjects per fold, of which roughly 4 subjects were from the ED group. Based on these splits, we ran a nested 5‐fold cross‐validation, that is, we trained our models on 4 folds, while testing it on the held‐out fold. During training, one of the four folds was additionally held out, based on which the hyperparameters of the respective model were optimised.

The models were trained on the total sum of instances in each training fold. This was equivalent to the number of utterances in each video (as we extracted one feature vector per utterance). However, the ground truth was available on the speaker level. This means we were able to treat the independent predictions obtained over all instances in a video as a *bag of predictions* which needed to be aggregated to provide one final, speaker‐level prediction, that is, we applied majority voting across the sample level predictions to obtain one prediction per speaker.

For our evaluation, we used the unweighted average recall (UAR; the average recall for each class; also referred to as *balanced accuracy* in literature). This we computed both on the instance‐level (UAR_I_) and the speaker‐level (UAR_SP_). See Kathan et al. (2023) for more details on this process.

## 3 Results

### 3.1 Sample Characteristics

A total of 44 mother‐child dyads were analysed in this study. *n* = 17 women had a history of ED (AN = 12, BN = 2, OSFED = 3) while *n* = 27 served as control group. At study inclusion, 10 women of the ED group had an active ED, while seven were remitted for an average of 9.71 ± 3.09 years. There were no differences between the groups regarding sociodemographic variables (Table 1). However, mothers with ED history reported significantly higher ED psychopathology (*Z* = −4.255; *r* = 0.64; *p* < 0.001), with Shape Concern being the most pronounced ED symptom. Moreover, women with ED history had significantly higher depression scores (PHQ‐9: *Z* = −2.596, *r* = 0.39, *p* = 0.009). There were no differences between the children of the two groups regarding age, birth weight and weight at 10 months (Table 1). For our machine learning analysis, we detected and analysed overall a total of 4528 utterances, lasting a total of 6.78 h, the majority of which resulting from the control group with 4.37 h, and the ED group only contributing 2.41 h in utterance length, which closely reflects the subject ratio between the two groups.

**TABLE 1:** Maternal and child characteristics.

	Total sample		ED		HC		ES	
	%	*n*	%	*n*	%	*n*	*p* value	*Φ*
Maternal characteristics								
Nullipara	61.4	27	58.8	10	63.0	17	1.000^c^	0.04
German nationality	93.2	41	100.0	17	88.9	24	0.272^c^	0.22
University degree	75.0	33	70.6	12	77.8	21	0.724^c^	−0.08
In a relationship	95.5	42	88.2	15	100.0	27	0.144^c^	0.28
Family income ≥ 4000	59.1	26	47.1	8	66.7	18	0.225^c^	0.19

	*M* ± SD	*N*	*M* ± SD	*n*	*M* ± SD	*n*	*p* value	*d/r*
Age (years)	31.32 ± 4.34	44	32.06 ± 4.10	17	30.85 ± 4.50	27	0.375^a^	0.28
BMI (kg/m^2^)	21.54 ± 2.86	43	21.03 ± 3.06	16	21.84 ± 2.75	27	0.375^a^	0.28
EDE‐Q								
Restraint	0.91 ± 1.37	44	1.91 ± 1.75	17	0.28 ± 0.40	27	**<** **0.001** ^b^	0.51
Eating concern	0.34 ± 0.57	44	0.73 ± 0.75	17	0.10 ± 0.15	27	**<** **0.001** ^b^	0.58
Weight concern	0.98 ± 1.25	44	1.95 ± 1.52	17	0.36 ± 0.42	27	**<** **0.001** ^b^	0.62
Shape concern	1.32 ± 1.33	44	2.36 ± 1.49	17	0.66 ± 0.64	27	**<** **0.001** ^b^	0.57
Total score	0.89 ± 1.00	44	1.74 ± 1.13	17	0.35 ± 0.28	27	**<** **0.001** ^b^	0.64
PHQ‐9	6.77 ± 4.69	44	9.35 ± 5.35	17	5.15 ± 3.41	27	**0.009** ^b^	0.39
PSS10	15.61 ± 7.01	44	17.00 ± 6.88	17	14.74 ± 7.07	27	0.303^a^	0.32
Child characteristics								
Age (months)	10.30 ± 0.46	43	10.21 ± 0.21	16	10.35 ± 0.56	27	0.220^a^	0.32
Birth weight (kg)	3.42 ± 0.46	44	3.31 ± 0.47	17	3.50 ± 0.45	27	0.228^b^	0.18
Weight at 10 months (kg)	9.69 ± 1.40	44	9.52 ± 1.46	17	9.80 ± 1.39	27	0.531^a^	0.20

*Note:* Data presented as mean (*M*) ± standard deviation (SD). Bold values indicate *p* < 0.05.

Abbreviations: BMI, body mass index; ED, eating disorder group; EDE‐Q, eating disorder examination questionnaire; HC, healthy control group; PHQ‐9, Patient Health Questionnaire‐9; PSS10, Perceived Stress Scale.

^a^ Student's *t*‐Test.

^b^ Mann‐Whitney‐U‐Test.

^c^ Fisher's Exact Test.

### 3.2 Machine Learning Analysis

The ML results for all experiments are reported in Table 2 in terms of the UAR, with the mean (and 95% Cl) over the five folds. We first observed that speech features extracted over a rolling window resulted in chance‐level performance (50%, Table 2). This indicated on the one hand that audio features struggled to obtain good performance, and that further segmenting individual utterances with rolling windows resulted in worse performance for this task. As hyperparameters were optimised per fold on the corresponding validation set, the specific settings are not further reported in the results.

**TABLE 2:** ML results with 95% Cl for speech parameters.

Features	UAR_I_ [%]	UAR_SP_ [%]
eGeMAPS [rolling]	50 [50; 50]	50 [50; 50]
MFCC [rolling]	46 [46; 47]	51 [45; 59]
eGeMAPS [utterance]	51 [50; 52]	50 [50; 50]
w2v2‐large	53 [52; 55]	58 [42; 73]
w2v2‐emo‐emb	51 [50; 53]	56 [41; 71]
w2v2‐emo‐avd	57 [55; 57]	59 [46; 70]

*Note:* Data presented as mean (*M*) results with 95% CI for a subject‐independent 5‐fold cross‐validation. Note that we did hyperparameter and classifier selection based on the development set on a fold‐level, which may lead to different settings being selected across folds.

Abbreviations: eGeMAPS, extended Geneva Minimalistic Acoustic Parameter Set; MFCC, Mel frequency cepstral coefficients; UAR_I_, unweighted average recall, instance‐level; UAR_SP_, unweighted average recall, speaker‐level.

Speech features extracted over the entire utterance resulted in moderately better performance—at least exceeding chance‐level. This was particularly true for learnt representations from w2v2 models, with the best performance of 59% obtained using the output predictions of w2v2‐emo (Table 2).

**FIGURE 2:**
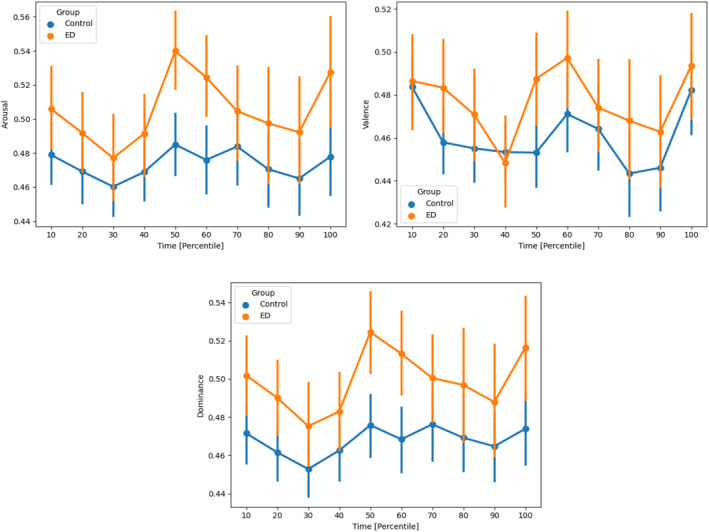
Arousal, valence and dominance in maternal speech throughout the videos.

Given that w2v2‐emo‐avd yielded the best results (though only marginally better than w2v2‐large), we proceeded to visualise the predictions of arousal, valence, and dominance for all participants in Figure 2. Specifically, we visualised the mean and 95% confidence intervals obtained with bootstrapping. Furthermore, these figures illustrated the AVD values for percentiles of video duration—for example, the 10^th^ percentile corresponded to the utterances that were recorded in the beginning 10% of the video. This allowed us to compare AVD values in time for videos of different duration.

The curves reveal interesting trends (Figure 2). First of all, we noted that the group of ED participants shows slightly higher arousal, valence, and dominance, throughout the feeding situation, with arousal and dominance showing larger differences than valence. However, statistical significance (*p* < 0.05) according to an analysis of variance (ANOVA) can only be reported for all emotional dimensions for the percentile 50 and for arousal and dominance for the percentile 60 and 100. All three dimensions hovered around neutral and tended to lean negative. Moreover, all three variables peaked in the middle of the interaction, with the peak being more pronounced for the ED group than the control (on average, this occurred approx. 6 min after the beginning of each video). In this stage, the separation between the two groups was at its highest, with no overlap in the confidence intervals.

## 4 Discussion

This study employed deep learning techniques to analyse maternal voice data, with a particular focus on affective dimensions in maternal speech during feeding situations in mothers with and without ED history and their children. While it was not possible to distinguish the groups based on the speech features, we found that maternal arousal, valence, and dominance were consistently stronger expressed in the voices of mothers of the ED group throughout the feeding situations, with statistical significance for all emotional dimensions for the percentile 50 and for arousal and dominance for the percentile 60 and 100. However, despite elevated arousal levels observed in the voices of the ED group, they maintained a neutral state, whereas the HC group demonstrated a tendency toward inactivity. Analogous outcomes as for arousal were observed for dominance. Concurrently, both groups showed a general inclination towards negative valence. Furthermore, a peak was observed in all three affective variables in the middle of the feeding interaction, with the peak being more pronounced for the ED group than the control group.

Arousal spans from a state of sleep, through varying levels of drowsiness and alertness, to intense excitement at the other extreme (Russell and Mehrabian 1977). Higher levels of arousal are indicative of a heightened level of activation, which may be associated with positive emotions such as happiness, but could also be indicative of negative emotions such as fear or anger (Gangamohan et al. 2019). In general, elevated levels of arousal suggest that the person perceives a situation as more stimulating or stressful. As has been previously reported in the literature, mealtimes in families with EDs are often problematic and characterised by conflict (Chapman et al. 2021). This suggests the possibility that heightened levels of arousal may be attributable to negative emotions experienced. Furthermore, this would align with data previously reported from our sample, showing considerable psychopathology in the ED group and difficulties in adjustment (Throm, Dörsam, et al. 2024). However, we did not find significant differences in the subjective stress scores as assessed by the PSS between the two groups. Further, when the elevated arousal scores are considered alongside the higher levels of valence, which reflects an individual's perception of pleasantness or unpleasantness (Stasak and et al. 2016), the findings rather suggest that mothers with ED history perceive the feeding situation as more positive and pleasant than mothers in the control group. Although this contradicts what we expected, it fits with a result found in a recent analysis of observational feeding data of a subsample of the EMKIE study by our group, which did not fully overlap with the sample in the present study. That study found that the quality of mother‐child interaction during feeding was not affected by ED psychopathology and that mothers in the control group made significantly more negative statements about their infant's food intake or preferences and were more concerned about mess during the feeding than mothers with ED history (Doersam et al. 2024). The authors concluded that the mother's concern about transmitting their disordered eating might be an explanation for those findings, in the form of an overcompensation and an attempt to achieve ‘optimal’ performance during feeding interactions (Doersam et al. 2024), which may be also applicable for the findings of the present study. It is important to note that despite having higher levels compared to mothers in the HC group, mothers in the ED group maintained within a ‘normal’ range of arousal. Both groups showed an inclination towards negative valence, a tendency that was more evident among the mothers in the HC group. This finding indicates that, despite exhibiting higher valence levels compared to those without ED, mothers in the ED group still perceived the situation as uncomfortable.

We refrained from interpreting the findings regarding dominance, as our analysis demonstrated a strong correlation with arousal. This finding indicates that the model may lack the capacity to differentiate between dominance and arousal, thereby precluding further conclusions regarding the dimension dominance.

Linking emotional dimensions to categoric dimensions is difficult, as mapping is not constant. However, a tentative explanation for the higher levels of arousal and valence in voices of mothers with ED history might also be attributed to their greater overall emotional involvement in the situation. As eating is a core component of eating disorders (Treasure et al. 2012), mealtimes can be emotionally significant experiences, potentially exerting a considerable influence on individuals currently or formerly affected by eating disorders. For mothers in the control group, mealtimes may be more unencumbered events that are of lesser importance than for women with ED, which may have resulted in lower emotional involvement or an emotional state of boredom. However, this has to be tested in further studies.

The peak in arousal and valence, with significant differences between the groups, shows that the interactions between mother‐child dyads exhibit ‘emotional hotspots’ that are clustered in the middle of the feeding interaction. It is possible that the mothers became accustomed to the unfamiliar situation of being filmed during mealtimes and may experience a sense of relief if the child is eating well, thereby enhancing the overall pleasantness of the situation. As time progresses, the children's appetite may diminishes, and they become more engaged with other activities than the food itself, impeding the eating situation with the result of reduced valence levels.

Reasons for the different results across ML approaches may lie in the way they process the data. While the models based on the traditional feature extraction methods (MFCC and eGeMAPS) are tasked to learn differences in the patterns solely from the comparatively small data set of our study, wav2vec is an already trained deep learning model that benefits from a large data basis, promising to leverage information through a rich emotional representation.

## 5 Strengths and Limitations

The main strength of this study is the pioneering use of ML approaches for the analysis of maternal voice in mealtime situations in families with and without ED history. This enables an objective evaluation in real time and minimises the risk of subjective judgement. Furthermore, the use of videotaped observations as a data source is advantageous over written texts as it captures spontaneous communication in a more authentic manner.

Nevertheless, some limitations have to be considered in the context of our results. Despite the amount of data being comparable to that of similar explorative analyses for voice patterns related to diseases and disorders, it is possible that patterns do exist, but are subtler, such that more data would be necessary to successfully train the ML algorithms or that the differences could only be discovered with different types of features or ML algorithms. Furthermore, the unstructured nature of the recording sessions and the general audio quality pose additional challenges to the ML experiments and thus might limit the expressiveness of the results. Finally, we note that any interpretation of AVD values is *tentative*. These are not variables annotated by human raters (or self‐reported by the subject themselves). Rather, they are predicted by an ML model trained on such data on one dataset (Wagner et al. 2023). This model necessarily inherits the biases present in that data, which potentially include, among others, biases with respect to gender, ethnicity, recording conditions, and the demographics of the raters used to annotate it. A further downside of the model is that it relies on linguistic information for its valence predictions (Triantafyllopoulos and et al. 2022). Given that it was trained on English data and the data in this study is German, there is a high degree of unreliability regarding this variable in particular. Nevertheless, we expect these errors to be systematic, that is the trend of the errors is consistent across all speakers and utterances. The observed differences across the groups may therefore indicate an underlying effect, even though the raw scores should be interpreted with more caution. A similarly performing model solely focused on the German language might overcome such limitations but is currently not available in literature. General limitations regarding the EMKIE study have been described elsewhere (Doersam et al. 2024).

## 6 Conclusions and Implications for Further Research

Although it was not possible to distinguish women with and without ED history on the basis of speech features, ML models revealed differences in the emotionality in mothers' voices during mealtimes with their children in women with and without ED history. Women with ED history were found to be more emotionally involved in the feeding situation, with arousal, valence, and dominance being consistently stronger expressed in the voices of those mothers. The middle of the mealtime situation seems to be an ‘emotional hotspot’ in mother‐child mealtime interaction, independent of ED diagnosis. The findings of this study highlight the importance of research into maternal communication in families with ED history, as differences in the affective dimensions in the maternal voices during feeding were found. This may be a mechanism in the transgenerational transmission of the disorder. The use of ML approaches is promising as they may detect more subtle nuances as compared to rating schemes or self‐report. Clinical implications of our findings include targeted preventive interventions focussing on mothers‐to‐be with ED and their children as particular risk groups for the development of mental health burden.

## Author Contributions

**Jana Katharina Throm:** conceptualization, formal analysis, investigation, writing – original draft, writing – review and editing, visualization, project administration. **Manuel Milling:** conceptualization, methodology, formal analysis, writing – original draft, writing – review and editing, visualization. **Andreas Triantafyllopoulos:** conceptualization, methodology, formal analysis, writing – original draft, writing – review and editing, visualization. **Alexander Kathan:** conceptualization, methodology, formal analysis, writing – original draft, writing – review and editing, visualization. **Annica Franziska Dörsam:** investigation, writing – review and editing, project administration, funding acquisition. **Johanna Löchner:** conceptualization, writing – review and editing. **Björn Schuller:** conceptualization, methodology, writing – review and editing, supervision. **Katrin Elisabeth Giel:** conceptualization, writing – review and editing, supervision, project administration, funding acquisition.

## Ethics Statement

This study was approved by the ethics committee of the medical faculty of the Eberhard‐Karls‐University and the University Hospital Tübingen (219/2018BO1; 859/2021BO2).

## Consent

All study participants analysed in this study provided written informed consent.

## Conflicts of Interest

The authors declare no conflicts of interest.

## Data Availability

The data that support the findings of this study are available from the corresponding author upon reasonable request.
